# A Practical Guide to Identify Patients With Multifocal Motor Neuropathy, a Treatable Immune-Mediated Neuropathy

**DOI:** 10.1016/j.mayocpiqo.2023.12.002

**Published:** 2024-01-08

**Authors:** Jeffrey A. Allen, Amy E. Clarke, Thomas Harbo

**Affiliations:** a Department of Neurology, University of Minnesota, Minneapolis, MN; b Optum, Eden Prairie, MN; c Department of Neurology, Aarhus University Hospital, Aarhus, DK

## Abstract

Multifocal motor neuropathy (MMN) is a rare immune-mediated motor neuropathy characterized by asymmetric weakness that preferentially affects distal upper limb muscles. The clinical features of MMN may be difficult to differentiate from motor neuron disease. Other conditions that may be mistaken for MMN include inclusion body myositis, chronic inflammatory demyelinating polyradiculoneuropathy, hereditary neuropathy with liability to pressure palsy, focal neuropathies, and radiculopathies. A key distinguishing electrophysiologic feature of MMN is the motor nerve conduction block located at noncompressible sites. MMN is a treatable neuropathy; therefore it is important that primary care physicians are aware of the features of the disease to identify potential patients and make referrals to a neuromuscular specialist in a timely manner. This review provides an overview of the disease, highlights key differential diagnoses, and describes available treatment options for patients with MMN.

**Article Highlights**

• Multifocal motor neuropathy (MMN) mostly affects the distal upper limbs and is characterized by asymmetric weakness along the distribution of the individual named nerves, unaccompanied by pain or sensory loss.
• The key distinguishing electrophysiologic feature of MMN is the motor nerve conduction block located at noncompressible sites.
• As MMN is a rare disease, it mimics other diseases, such as amyotrophic lateral sclerosis, chronic inflammatory demyelinating polyradiculoneuropathy, hereditary neuropathy with liability to pressure palsy, inclusion body myositis, focal neuropathies, and radiculopathies, which may be suspected before the identification of MMN.
• The pattern of weakness, absence of sensory findings or pain, and conduction block at noncompressible sites are key features that can raise suspicion of MMN.
• When MMN is considered, referrals to a neuromuscular center with expertise in the diagnosis and treatment of rare inflammatory neuropathies are encouraged (https://www.gbs-cidp.org/support/centers-of-excellence/).

Multifocal motor neuropathy (MMN) is a rare, immune-mediated neuropathy with a prevalence of less than 1 in 100,000.^1^^,^^2^ As with many rare diseases, recognizing characteristic clinical features of the disease can be challenging, especially when other disorders with overlapping signs and symptoms are far more common. In a study of 46 patients who eventually turned out to have MMN at a neuromuscular center, only 6 had been referred for suspected MMN.^3^ The MMN diagnostic delay is common, often taking more than a year before the diagnosis is confirmed.^3^^,^^4^ When the diagnosis is delayed, treatment is as well, which may lead to missed opportunities to prevent the accumulation of irreversible neurologic impairment and disability.^4^^,^^5^

MMN-associated weakness is often attributed to other conditions before a diagnosis of MMN is established. Motor neuron disease, or amyotrophic lateral sclerosis (ALS), is one common MMN mimic.^4^ Unlike ALS, in which median survival is 3-5 years after symptom onset, MMN is a treatable, non-life-threatening disease with a normal life expectancy.^4^ Other conditions with different prognostic and treatment opportunities that may be mistaken for MMN include inclusion body myositis (IBM), chronic inflammatory demyelinating polyradiculoneuropathy (CIDP), focal or entrapment neuropathies, and radiculopathies.^6^^,^^7^ Furthermore, because of the frequency with which focal neuropathies and radiculopathies occur, they represent an important group of conditions to highlight.

The implications of misdiagnosis may be substantial, potentially exposing patients to inappropriate medications or surgeries, delaying the initiation of disability-modifying MMN treatment, and placing an emotional burden on patients that can detrimentally affect the quality of life. The intent of this review is to differentiate the key features of MMN from more common mimics to help clinicians who do not routinely encounter MMN in their practice identify patients who may benefit from an expeditious referral to a neuromuscular specialist.

### MMN: Key Clinical and Laboratory Features

MMN affects men more commonly than women, with a ratio of about 3:1.^8^ Disease onset for most patients is in the fourth or fifth decade of life.^1^^,^^2^ In 80% of patients, weakness first develops in the upper limbs and preferentially targets distal muscles.^3^ Approximately 20% of patients experience the onset of weakness in the lower limbs.^3^ Weakness is typically focal and asymmetric, following the distribution of individual named nerves.^6^ After onset, some patients experience slowly progressive worsening, whereas others can have stepwise or abrupt deteriorations as new motor nerves become affected.^6^ Although ∼20% of patients with MMN report mild distal lower limb sensory changes, sensory loss and neuropathic pain are otherwise not part of the MMN clinical spectrum.^2^^,^^6^ Cramps and fasciculations may occur in the affected limbs of up to 40% of patients.^2^ Cranial nerve involvement is distinctly unusual. On neurologic examination, deep tendon reflexes may be reduced in the affected limb (but intact in areas without weakness) and pathologic upper motor neuron signs should not be present.^6^

MMN is thought to be caused by dysfunction at the node of Ranvier rather than in the myelin or axon.^2^ Nodal dysfunction prevents propagation of electrical impulses down the course of a nerve, which in turn, leads to the classical MMN electrophysiological finding on nerve conduction studies (NCS) of conduction block (CB).^2^ Electrophysiologic studies may also reveal conduction velocity slowing and prolonged minimum F-wave latency.^1^ CB is not always appreciated in patients who otherwise have a strong clinical case for MMN.^1^^,^^2^ Failure to detect CB can occur when it is located in proximal nerve segments.^1^ In patients with long-standing disease, axon loss can lead to muscle atrophy on examination and loss of compound muscle action potential amplitude on NCS.^4^^,^^7^ When compound muscle action potential amplitudes are very small, CB may also be difficult to detect.^4^^,^^7^ In these patients with equivocal electrophysiologic changes, laboratory testing can help support the diagnosis of MMN.

Anti-monosialotetrahexosylganglioside (anti-GM1) immunoglobulin (Ig)M antibodies may be detected in at least 40% of patients with MMN.^9^ Anti-GM1-positive patients more often have severe weakness, disability, and eventual axon loss than seronegative patients.^9^ It has been suggested that anti-GM1 antibodies induce complement-mediated injury at the nodes of Ranvier, which leads to CB and weakness.^2^ Thickened nerves on magnetic resonance imaging or ultrasound or objective responses to treatment with intravenous immunoglobulin (IVIG) also supports the diagnosis of MMN.^2^^,^^6^

### MMN Burden of Disease

Disability in MMN can be variable. Because of the preferential involvement of the distal upper limbs, most patients develop restrictions related to fine motor skills of the hand, causing limitations with household, professional, and leisure activities.^10^ Distal lower limb weakness can also limit walking ability.^3^ An important predictor of irreversible disability is the development and accumulation of axon loss, which can be exacerbated when the diagnosis and initiation of treatment are delayed.^5^^,^^10^ Although the physical burdens of the disease are apparent, there is also a psychological burden that may not be as noticeable. In 1 cohort of 17 patients with MMN, overall quality of life was reduced.^11^ Physical domains were the most affected, but psychological aspects were also affected, especially for patients with a longer disease duration.^11^ Economic burden because of the cost of treatment or lost employment may add to the psychological burden of the disease. Such factors vary greatly, depending on the health care system of the country or region where patients receive treatment, available support networks, and travel costs to sites of care.

### Diagnosis of MMN

The European Federation of Neurological Societies/Peripheral Nerve Society guidelines for MMN, previously updated in 2010, propose diagnostic criteria for MMN. The core clinical criteria required for MMN diagnosis are as follows: (1) slowly progressive or stepwise progressive, focal, asymmetric limb weakness in the motor nerve distribution of at least 2 nerves for more than 1 month; and (2) no objective sensory abnormalities, except for minor vibration sense abnormalities in the lower limbs.^6^ Exclusion criteria for the diagnosis of MMN include the presence of upper motor neuron (UMN) signs, marked bulbar involvement, marked sensory loss, and symmetric weakness during the initial weeks of disease.^6^ Motor CB is the main electrophysiological criterion in the diagnosis of MMN. Although CB is most commonly found in mid-forearm nerves, it may also be present in more proximal areas that are difficult to assess with NCS.^6^ For CB to support a diagnosis of MMN, its location must not be at common sites of compression (eg, median nerve at the wrist or ulnar nerve at the elbow).^6^ If weakness and CB at noncompressible sites are present in 2 or more nerve distributions, then a diagnosis of MMN can be strongly considered.^6^ For specific descriptions of definite and probable motor CB, please refer to the published guidelines.^6^

### Differential Diagnoses

In Table 1, we provide an overview of the key clinical features for the differential diagnoses of MMN. Figure 1 presents a general approach that clinicians may take to identify patients with a motor neuropathy.

**Table 1:** Clinical Features of Differential Diagnoses of MMN^5^^,^^12^^,^^14^^,^^17^, ^18^, ^19^, ^20^, ^21^, ^22^

	Typical Pattern of Weakness			Sensory Loss
	Symmetry	Proximal/Distal	Limb Predominance	
MMN	Asymmetric	Distal > Proximal	UL	No
ALS (LMN variants)				
Flail arm	Symmetric	Proximal > Distal	UL	No
Flail leg	Asymmetric	Distal > Proximal	LL	No
Multifocal CIDP	Asymmetric	Distal > Proximal	UL or LL	Yes
Motor CIDP	Symmetric	Distal and proximal	UL or LL	No
HNPP	Asymmetric	Recurrent focal mononeuropathies at common sites of nerve compression		Yes
IBM	Asymmetric	Finger/wrist flexor and knee extensor muscles, sometimes with dysphagia		No
Hirayama disease	Asymmetric	Distal > Proximal	UL	No

Clinical features	
Focal neuropathies	Often occur due to nerve compression at common sites. Confirmed with electrodiagnostic tests showing CB.
Median neuropathy at wrist (ie, carpal tunnel syndrome)	• Pain, paresthesia, and sensory loss in the hand • Weakness of thumb abduction and opposition may develop after sensory loss
Ulnar neuropathy at the elbow (ie, cubital tunnel)	• Pain, paresthesia, and sensory loss in digit 4 and digit 5 of the hand • Weakness of the intrinsic hand muscles and grip weakness may develop after sensory loss
Radial neuropathy at the spiral groove (ie, Saturday night palsy)	• Abnormal sensory changes in the dorsum of the hand and along the posterior aspect of the arm • Weakness in finger extensors and wrist extensors
Peroneal neuropathy at the fibular head	• Often presents as foot drop that can range from a minor dragging of the toes to a steppage gait • Pain, numbness, paresthesia, and sensory loss may be present in the outer portion of the calf and top of the foot
Radiculopathies	
Cervical radiculopathies	• Commonly present as unilateral neck pain with radiation into the arm of the same side • Muscle weakness and sensory loss can manifest across varying locations down the arm
Lumbosacral radiculopathies	• Commonly present as unilateral low back pain with radiation into the leg of the same side • Muscle weakness and sensory loss can manifest across varying locations down the leg

Abbreviations: ALS, amyotrophic lateral sclerosis; CB, conduction block; CIDP, chronic inflammatory demyelinating polyradiculoneuropathy; HNPP, hereditary neuropathy with liability to pressure palsy; IBM, inclusion body myositis; LL, lower limb; LMN, lower motor neuron; MMN, multifocal motor neuropathy; UL, upper limb.

Adapted from Garg et al^12^ and Lawson et al.^5^

**Figure 1:**
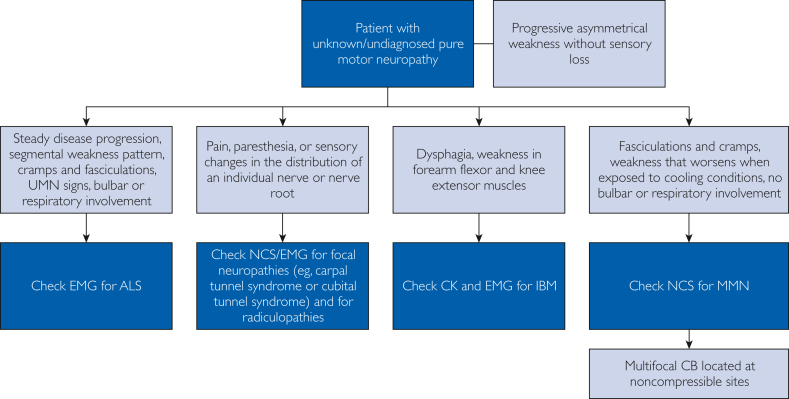
General approach for the identification of patients with motor neuropathy. ALS, amyotrophic lateral sclerosis; CB, conduction block; CK, creatine kinase; EMG, electromyography; IBM, inclusion body myositis; NCS, nerve conduction studies; MMN, multifocal motor neuropathy; UMN, upper motor neuron.

#### Amyotrophic Lateral Sclerosis

ALS is a degenerative disorder of motor neurons that presents clinically as asymmetric weakness without sensory loss.^4^^,^^12^ In its classic form, ALS is characterized by UMN and lower motor neuron (LMN) signs and symptoms in the same limb or body segment, with multiple body segments (bulbar, cervical, thoracic, and lumbosacral) that are affected as the disease progresses.^4^^,^^12^ Some patients with ALS have more restricted forms of the disease, which may include predominantly UMN or predominantly LMN involvement, whereas others can develop symptoms that are limited to the bulbar (progressive bulbar palsy), cervical (flail arm syndrome), or lumbosacral (flail leg syndrome) segments.^12^

In contrast to the multifocal pattern of weakness in MMN that follows named nerve distributions, weakness in ALS follows a distribution that can be mapped to spinal cord segments.^4^^,^^6^ Weakness in ALS typically progresses in a predictable pattern as contiguous spinal cord segments become affected.^4^^,^^5^ In MMN, weakness may progress in a stepwise manner with periods of stable disease and unpredictable spreading from nerve to nerve.^4^^,^^6^ On examination, most patients with ALS present with UMN signs (hyperreflexia), whereas in MMN, reflexes tend to be absent or reduced.^4^^,^^6^ LMN signs (atrophy and fasciculations) are present in both conditions but are more prominent and widespread in ALS.^4^ Bulbar (dysarthria or dysphagia) and respiratory involvement are common in ALS but infrequent in MMN.^4^

Although the presence of UMN signs strongly favors a diagnosis of ALS, not all forms of ALS have UMN involvement.^4^ The LMN ALS variants can be more difficult to differentiate from MMN.^4^ For these patients, the pattern of weakness and electrophysiological features play a key diagnostic role: MMN weakness is along the named nerve distributions and usually has CB on electrophysiologic testing, whereas in ALS weakness is segmental and electrophysiologic testing shows denervating and reinnervating changes without CB.^4^^,^^6^ If CB is not found but suspicion of MMN remains, imaging with an MRI or ultrasound may be helpful.^2^^,^^6^ The presence of nerve enlargement or enhancement in nerve segments that are clinically affected would favor a MMN diagnosis, whereas their absence may more likely suggest ALS.^2^

#### Spinal Muscular Atrophy

Spinal muscular atrophy (SMA) comprises a group of hereditary diseases caused by damaged motor neurons.^12^ Although disease onset occurs very early in life for most patients (ie, SMA types I, II, and III), those with SMA IV may present in early adulthood.^12^ Unlike MMN, weakness in SMA IV preferentially affects the lower limbs in a relatively symmetric proximal pattern.^12^ Bulbar dysfunction may also occur.^12^ Both NCS and electromyography (EMG) in SMA show active denervation and chronic reinnervation changes, rather than multifocal CB.^13^ In suspected cases of SMA, identification of an *SMN1* sequence variation with genetic testing may be diagnostic.^12^

#### Inclusion Body Myositis

IBM is an acquired muscle disorder that, similar to MMN, presents as painless and asymmetric weakness predominantly affecting men in mid and late life.^14^ The pattern of weakness plays a key role in IBM diagnosis, as it characteristically affects the finger or wrist flexor muscles and knee extensor muscles early in the disease course.^14^ Dysphagia, which is unusual in MMN, may be involved in between 40% and 80% of patients with IBM.^14^^,^^15^ Creatine kinase is typically elevated (≤15 × upper limit of normal) in IBM but is normal or only minimally elevated in MMN.^14^ IBM is a chronic active myopathy; therefore, EMG can be an important tool to distinguish IBM from MMN.^14^

#### Chronic Inflammatory Demyelinating Polyradiculoneuropathy

CIDP is an immune-mediated neuropathy that causes both numbness and weakness.^1^^,^^5^ Multifocal CIDP, a variant of CIDP, is similar to MMN in that it also manifests as asymmetrical weakness and may follow the distribution of individual named nerves. The differentiating clinical and electrophysiological feature between these 2 neuropathies is the presence of sensory involvement.^5^^,^^16^ Although CB as a key electrophysiological feature can be present in both MMN and CIDP, there is usually a lack of other demyelinating changes in MMN. In CIDP, widespread demyelinating changes including distal latency prolongation and slowing of conduction velocity are required.^2^^,^^16^ Cold paresis, where patients experience worsening of weakness in cold conditions, is a common symptom of MMN and not frequently seen in patients with CIDP.^17^ Cerebrospinal fluid (CSF) protein analyses can be considered in patients with CIDP or MMN; patients with MMN tend to have normal CSF protein levels, while patients with CIDP often have elevated CSF protein levels.^5^^,^^16^ Also, CIDP and MMN differ in prognosis and treatment paradigms. While both conditions respond to IVIG, unlike patients with CIDP, those with MMN may paradoxically worsen when exposed to corticosteroids and plasma exchange.^5^^,^^16^

#### Hereditary Neuropathies

Hereditary neuropathy with liability to pressure palsy (HNPP) is an uncommon mimic of MMN but an important consideration in certain populations.^2^^,^^6^ Unlike with MMN, where onset before 40 years of age is unusual, with HNPP, symptoms often manifest in the second or third decades of life.^18^ Similar to patients with MMN, those with HNPP present with asymmetric weakness and can have CB on electrodiagnostic studies.^5^^,^^18^ Although these features overlap with MMN, CB in HNPP is often present at common sites of nerve compression.^18^ Clinical and electrophysiological evidence of sensory loss is also present in HNPP.^18^ Approximately 80%-90% of patients with HNPP have a deletion of the *PMP22* gene.^18^ Other genetically determined mimics of MMN may include hereditary motor neuropathies. When HNPP is suspected in patients who develop progressive weakness predominantly affecting the lower limbs, with an onset before 20 years of age, genetic testing can be diagnostic.^12^

#### Hirayama Disease

Hirayama disease is a rare disease that clinically manifests in a similar manner to MMN. Patients with Hirayama disease are more commonly men and present with unilateral or asymmetric distal weakness in the upper extremities with no sensory loss.^19^ Characteristics that can discriminate Hirayama disease from MMN include the age of onset, as Hirayama often appears during puberty (12-20 years of age), and imaging that shows asymmetric cord flattening and lower cervical cord atrophy.^19^

#### Focal Neuropathies

Focal nerve entrapment or compression may be mistaken for MMN, especially in the early stages of the disease. Median neuropathy at the wrist (ie, carpal tunnel syndrome), ulnar neuropathy at the elbow (ie, cubital tunnel syndrome), radial neuropathy at the spiral groove (ie, Saturday night palsy), and peroneal neuropathy across the fibular head are the most commonly encountered focal neuropathies.^20^^,^^21^ Sensory symptoms and pain are common in these conditions. The absence of sensory findings may raise the possibility of MMN.^3^^,^^6^^,^^20^ NCS are also critically important for diagnosis. The location of CB in focal neuropathies is always across the site of compression or entrapment, but adjacent nerve segments should be normal.^20^^,^^21^ If CB is present in a motor nerve at a noncompressible site (eg, in the forearm rather than across the wrist or across the elbow) and sensory nerve conduction is normal, then suspicion of MMN is heightened.^6^ Other features of MMN, including fasciculations, cramps, and reduced reflexes, are also typically not appreciated in focal neuropathies.^6^^,^^21^

#### Radiculopathies

Cervical and lumbosacral radiculopathies may present with asymmetric weakness and diminished or absent reflexes in the affected limb.^22^^,^^23^ However, patients with radiculopathies also experience radiating neck or back pain and sensory disturbances in the affected limb.^22^^,^^23^ The distribution of weakness and numbness follows nerve root distributions as opposed to the individual motor nerve pattern in MMN.^6^^,^^22^^,^^23^ Both NCS and EMG play an important diagnostic role for radiculopathy.^23^ While both conditions can have abnormal motor NCS and normal sensory NCS, in radiculopathy the denervation and reinnervation changes may be mapped to a nerve root rather than a specific nerve distribution, and CB is not present.^6^^,^^23^ MRI can be used to view the cervical and lumbosacral spines to assess the source of impingement.^22^^,^^23^

### Treatment and Management of MMN

IVIG is first-line treatment for patients with MMN.^2^ Efficacy is established on the basis of several randomized clinical trials that have reported improved muscle strength and amelioration of disability in treated patients.^6^ These results have informed MMN treatment guidelines, which advise induction therapy with IVIG 2 g/kg administered over 2-5 days and maintenance therapy with IVIG 1 g/kg every 2-4 weeks or 2 g/kg every 1-2 months.^6^

Subcutaneous immunoglobulin (SCIG) has also been investigated for the treatment of patients with MMN.^6^ For those with MMN who are IVIG-responsive, SCIG is shown to be as safe, tolerable, and equally effective as IVIG.^24^^,^^25^ Unlike other immune-mediated neuropathies, corticosteroids and plasma exchange are usually not effective and may worsen weakness in patients with MMN.^6^

Adherence to accepted standards of practice, such as those provided by the Immunoglobulin National Society for Ig therapy in the United States, is vital to optimizing clinical outcomes for patients receiving Ig therapy.^26^ Strategies to mitigate IVIG-associated adverse events include completion of a risk assessment by a certified Ig pharmacist, assessment of comorbidities, proper hydration, and appropriate infusion rate titration.^26^ For SCIG, ensuring appropriate needle size, number of infusion sites, and administration technique is critical to optimizing patients’ experience.^26^

Other than Ig, there are no proven effective therapies for MMN.^2^ Although some case reports or case series have reported benefits with immunosuppressant therapies such as cyclophosphamide and mycophenolate, the risks for each treatment must be weighed against the uncertain benefits.^27^ For example, an uncontrolled study of 6 patients with MMN treated with oral cyclophosphamide reported improvements in Rankin disability scores, upper and lower limb impairment scores, and muscle strength.^28^ However, because of the toxicity associated with cyclophosphamide and the lack of evidence surrounding its efficacy in a controlled setting, guidelines do not recommend it as a desirable treatment option for patients with MMN.^6^

### Summary and Implications for Primary Care

MMN is characterized by asymmetric weakness without pain or sensory loss.^6^ The pattern of weakness follows the distribution of individual named nerves and most commonly affects the distal upper limbs.^2^^,^^3^^,^^6^ Mimics of MMN may include ALS, CIDP, HNPP, IBM, focal neuropathies, and radiculopathies.^6^^,^^7^ The pattern of weakness, absence of sensory findings or pain, and CB on electrophysiological testing at noncompressible sites are key features that raise suspicion of MMN.^6^ When a diagnosis of MMN is considered, we encourage referrals to neuromuscular centers with expertise in the diagnosis and treatment of rare inflammatory neuropathies (https://www.gbs-cidp.org/support/centers-of-excellence/).

## Potential Competing Interests

**Jeffrey A. Allen:** Consultant for Argenx, Alnylam, Alexion, Annexon, CSL Behring, Grifols, Takeda, Immunovant, Immupharma, Pfizer. **Amy E. Clarke:** Nothing to disclose. **Thomas Harbo:** Consultant for Argenx, Annexon, Janssen
